# Association between household environmental conditions and nutritional status of women of childbearing age in Nigeria

**DOI:** 10.1371/journal.pone.0243356

**Published:** 2020-12-11

**Authors:** Oyewale Mayowa Morakinyo, Ayo Stephen Adebowale, Taiwo Akinyode Obembe, Elizabeth Omoladun Oloruntoba

**Affiliations:** 1 Faculty of Public Health, Department of Environmental Health Sciences, College of Medicine, University of Ibadan, Ibadan, Nigeria; 2 Faculty of Public Health, Department of Epidemiology and Medical Statistics, College of Medicine, University of Ibadan, Ibadan, Nigeria; 3 Faculty of Public Health, Department of Health Policy and Management, College of Medicine, University of Ibadan, Ibadan, Nigeria; University of Cape Coast, GHANA

## Abstract

Maternal undernutrition remains a leading cause of morbidity and mortality in Nigeria. Yet, most interventional programmes are focused on infant and child nutrition outcomes and not on maternal nutrition‐related outcomes. Evidence suggests that the integration of household environmental interventions into nutrition actions can make a difference in reducing the burden of maternal undernutrition. This study examined the influence of household environmental conditions (HHEC) on the nutritional status of women of childbearing age in Nigeria using secondary data from the 2013 Nigeria Demographic and Health Survey. The original sample of 38,948 women age 15–49 years was selected using multi-stage probability sampling. The sample for the current analysis was 23,344 after exclusion of women due to health status or provision of incomplete information. The dependent and main independent variables were undernutrition (defined as Body Mass Index below 18.5) and HHEC (generated from cooking fuel, toilet type, source of drinking water, and housing materials) respectively. Data were analysed using descriptive statistics, Chi-square, and logistic regression model at 5% level of significance. The prevalence of undernutrition among women living in houses with unimproved and improved HHEC was 17.2% and 7.2% respectively. The adjusted odds of undernutrition was significantly higher among women who lived in houses with unimproved HHEC (aOR = 2.02, C.I = 1.37–2.97, p <0.001). The odds of undernutrition are greater in young women (aOR = 2.38, C.I. = 1.88–3.00, p <0.001) compared to older, and those of lower wealth status (aOR = 2.14, CI = 1.69–2.71, p <0.001) compared to higher. Other predictors of undernutrition in women of reproductive age in Nigeria include the level of education, marital status, and working status. Living in a house with unimproved environmental conditions is a predictor of undernutrition in women. The integration of environmental and nutrition programmes could assist in addressing this burden in Nigeria.

## Introduction

Malnutrition is a complex issue and remains the main cause of death and disease in the world [1]. Maternal undernutrition is a key determinant of adverse health outcomes for mothers and their offspring in both developed and developing countries [2]. The burden of poor maternal nutritional status is evident in the high global infant and maternal morbidity and mortality [3,4]. Annually, more than 3.5 million mothers and under-five children in South-central Asia and sub-Saharan Africa lose their lives or are permanently disabled from the psychological or underlying causes of undernutrition [3]. Underweight mothers are significantly more likely to have stunted children and this predisposes such children to high morbidity and mortality [5].

Although the prevalence of maternal undernutrition has been on the decline in recent times, nonetheless, high prevalence is still noticeable in sub-Saharan Africa [6]. Maternal undernutrition ranges between 10 and 40% in most countries in sub-Saharan Africa [7]. The findings from the year 2018 Nigeria National Nutrition and Health Survey (NNHS) indicated that 6.9 percent of Nigerian women age 15–49 years were acutely malnourished and 3.8 percent were severely malnourished [8].

**Fig 1:**
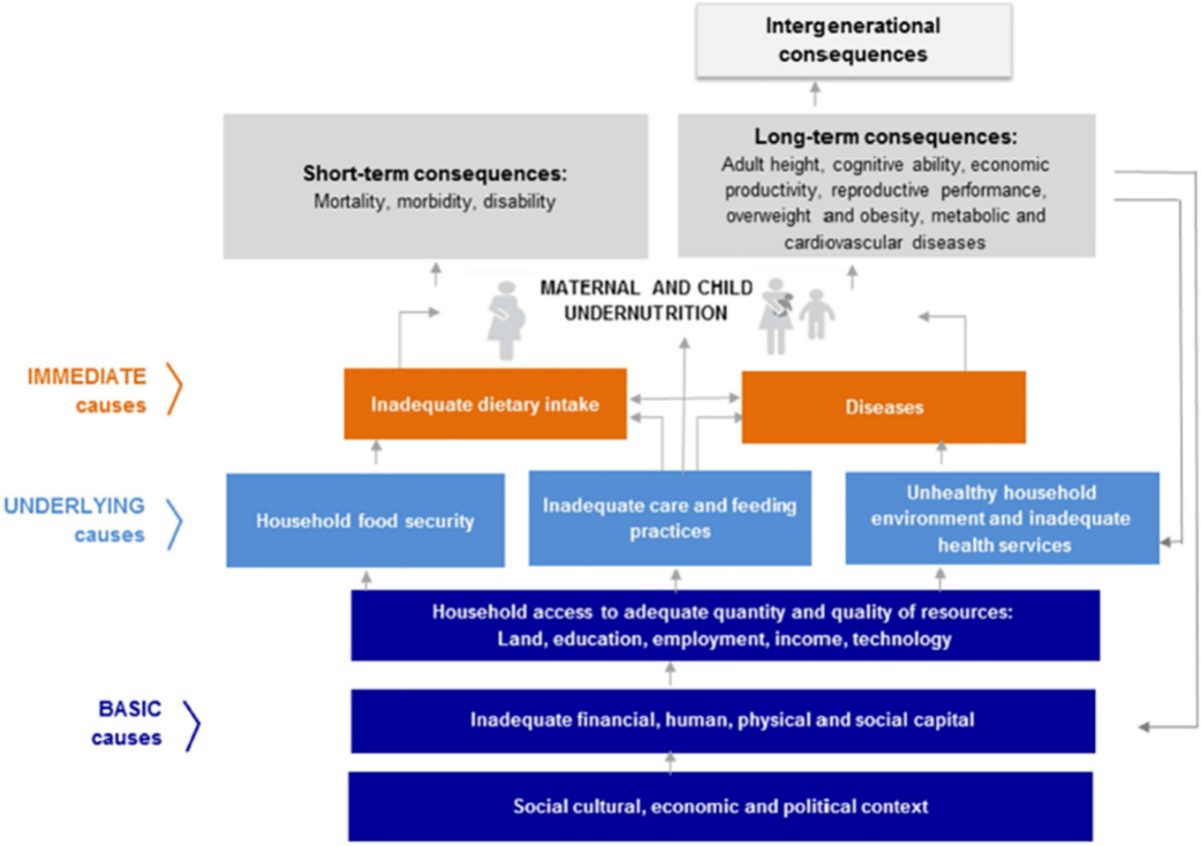
Conceptual framework of determinants of undernutrition [11].

The causes of undernutrition are well known and multifaceted [9]. The United Nations Children's Fund (UNICEF) conceptual framework (Fig 1) for the causes of malnutrition offers an interface between broader systemic level factors and community, household, and individual levels [10]. The framework clearly shows that malnutrition is a product of multiple and interrelated factors. These factors are immediate, underlying and basic, and can influence one another [10].

Unsuitable or insufficient food intake, poor care practices and disease, which are the three main underlying causes of undernutrition are directly or indirectly linked to insufficient access to water, sanitation, and hygiene (WASH) [12]. The World Health Organization (WHO) reported that an estimated 50% (39–61%) of the global burden of malnutrition is linked to the environment, particularly poor WASH conditions [13]. Poor WASH conditions promote the ingestion of faecal pathogens which causes diarrhoea, intestinal worms and environmental enteric dysfunction [14]. A good WASH environment is vital to shaping maternal and child nutritional outcomes [15]. The integration of WASH and nutrition into national policies and development partners’ strategies is on the rise in many countries who have realised the importance of adequate environmental conditions to achieve good nutritional status [15].

There is a renewed commitment by different stakeholders on ending the burden of undernutrition. The adoption of the Sustainable Development Goals (SDGs), the United Nations’ proclamation of a Decade of Action on Nutrition (2016–2025) and the Rome Declaration on Nutrition and the Framework for Action in the year 2014 by world leaders, suggested that well thought out and clear-cut actions are needed to tackle undernutrition [16]. Addressing undernutrition in all its forms will play a significant role in achieving goals 1 to 3 of the SDGs and other global targets relating to non-communicable diseases [17].

Moreover, the Scaling-Up Nutrition (SUN) movement among countries to achieve the multi-sectoral framework required to bring about sustainable nutrition-specific and nutrition-sensitive interventions further highlights the commitments to addressing the fundamental causes of undernutrition [18]. The Federal Republic of Nigeria joined the SUN Movement in the year 2011 having realised the importance of nutrition as a development issue and had committed to addressing malnutrition with the adoption of a National Health Strategic Development Plan [19]. Although the year 2018 progress report stated that most SUN countries are lagging in achieving key indicators, the movement has moved on to assessing how it can use its network to spur global progress on nutrition [18]. In Nigeria, inadequate budget and lack of a political will have often crippled the implementation of nutrition activities at the federal, state and local government levels [19].

The understanding of the different pathways to adequate nutrition among women of childbearing age is of utmost importance. With environmental factors being the root cause of most global disease burden, there is a need for a holistic and unified approach to the protection of public health. The integration of household environmental interventions into nutrition actions could likely help reduce the burden of maternal undernutrition in Nigeria.

However, in Nigeria, there is a paucity of information on how household environmental conditions as a composite unit can influence maternal undernutrition. This study seeks to advance existing knowledge by examining the influence of household environmental conditions on the nutritional status of women of childbearing age in Nigeria.

## Methods

### Ethical consideration

Ethical approval was obtained from the National Ethical Review Board of the Federal Ministry of Health before conducting this survey by the data originators (NHREC/2008/07). Written informed consent was obtained from women of reproductive age (15–49 years) at the point of data collection and were assured of the confidentiality and anonymity of the information provided.

### Sources of data and sampling

This cross-sectional design study involved analysis of secondary data collected on women during the 2013 Nigeria Demographic and Health Survey (NDHS).

The survey was carried out to provide reliable information about maternal and child health and family planning services in the urban and rural areas, across the country’s six geographical zones, and each of the 36 states and the Federal Capital Territory. For this nationally representative sample survey that covered the entire population residing in non-institutional dwelling units in Nigeria, a multi-stage probability sampling approach was used to select the eligible respondents, who were women of reproductive age (15–49 years). The sampling frame used was the list of enumeration areas (EAs). The primary sampling unit (PSU), referred to as a cluster in the survey, was defined based on EAs and the sample was selected using a stratified three-stage cluster design consisting of 904 clusters, 372 in urban areas and 532 in rural areas. A representative sample of 40,680 households was selected for the survey [20]. The eligible women that were interviewed were either permanent residents of selected households or visitors that were present in the households on the night before the survey. The household and eligible women’s response rates for the 2013 NDHS were 99.0% and 97.6% respectively [20].

The Global Positioning System (GPS) receivers were used in calculating the coordinates of the sampled clusters. A fixed sample take of 45 households was selected in every urban and rural cluster through equal probability systematic sampling. The overall sample size was 38,948 and this number reduced to 23,344 when women with incomplete information on the variables that were used to generate the main independent variable (Household Environmental Conditions) and those who were either ‘breastfeeding’ or ‘pregnant’ at the period of the survey were excluded from the sample. Exclusion of these women was to reduce bias that may be introduced as a result of loss of weight due to breastfeeding, and weight gain or weight loss because of the fetus for pregnant women. In this survey, 12.1% (4710) and 25.4% (9909) were currently pregnant and currently breast-feeding respectively. Only 1.5% (600) did not have information on either their weight or height, thus making the computation of body mass index impossible and 0.9% (384) have missing information in one or more of the variables used for the generation of household environmental conditions. The characteristics of women excluded did not differ significantly from the remaining sample. We therefore regard the resulting sample as being representative of the general population.

### Variable description

Anthropometry is a universally acceptable, inexpensive and non-invasive technique that is useful for the evaluation of physical growth and the nutritional status of individuals. The dependent variable was the Body Mass Index (BMI). It was used as a measure of nutritional status following the WHO stipulated guidelines [21]. This variable was created from the information on the weight and height of women. Weight was measured in kilogrammes by trained field workers using a calibrated weighing balance with a capacity of 200 kg to the nearest 0.1 kg. Height was measured in meter to the nearest 1 cm using a tape rule. Thus, the BMI was determined using the formula; *BMI* = *Weight*/(*Height*)^2^ [21]. The nutritional status was obtained as follows; Underweight if BMI<18.5kg/m^2^, Normal if 18.5kg/m^2^≤BMI<25.0kg/m^2^, Overweight if 25.0kg/m^2^≤BMI<30.0kg/m^2^ and Obesity if BMI≥30.0kg/m^2^ [21]. The analysis reported in this study was based on the underweight category.

The main independent variable was the Household Environmental Conditions (HHEC). This variable was generated from information on; cooking fuel, toilet type, source of drinking water, and housing materials. The classification of the variable HHEC was based on the information found in the literature on the association between cooking fuel, toilet type, source of drinking water, housing materials, and undernutrition [9,15,22,23]. In the original questionnaire used for the study, respondents were asked to select the main type of; cooking fuel, toilet type, source of drinking water, and main floor material, main roof material, main wall material from the list of options in Table 1. The categories of environmental factors shown in Table 1 were adapted from the groupings of variables from the year 2013 Nigeria National Demographic Health Survey and the year 2010 WHO and UNICEF report on sanitation and drinking water [20,24].

**Table 1:** Independent variables from the 2013 Nigeria Demographic and Health Survey.

HHEC	Categories as indicated in the questionnaire	Re-categorized		
Cooking fuel	Electricity, LPG, natural gas, biogas, kerosene, coal lignite, charcoal, wood, straw/shrub-grass, agricultural crop, animal dung, and others	**Biomass:** coal lignite, charcoal, wood, straw/shrub-grass, agricultural crop, animal dung, and others. **Clean fuel:** Electricity, LPG, natural gas, biogas, kerosene.	Biomass Clean Fuel	= 0 = 1
Toilet type	Flush to a pipe sewage system, flush to a septic tank, flush to a pit latrine, flush to somewhere else, flush but don’t know where it goes, ventilated improved pit latrine, pit latrine with slab, pit latrine without slab/open pit, no facility/bush/field, composting toilet, bucket toilet, hanging toilet/latrine, and others.	**Unimproved:** flush/pour flush not to sewer/septic tank/pit latrine, pit latrine without slab/open pit, bucket, hanging toilet/latrine, no facility/bush/field **Improved:** flush/pour flush to a piped sewer system, septic tank or pit latrine, ventilated improved pit latrine, pit latrine with slab, composting toilet	Unimproved Improved	= 0 = 1
Source of drinking water	Piped into dwelling, piped to yard/plot, public tap/standpipe, tube well or borehole, protected well, unprotected well, protected spring, unprotected spring, river/dam/lake/ponds/stream/canal/irrigation channel, rainwater, tanker truck, cart with small tank, bottled water, sachet water (in a bag), and others	**Unimproved:** unprotected well and spring, tanker truck/cart with drum, surface water, sachet water, and other sources **Improved:** piped into dwelling/yard/plot, public tap/standpipe, tube-well or borehole, protected well and spring, rainwater, and bottled water	Unimproved Improved	= 0 = 1
Main floor material	Earth/ sand, Dung, Wood planks, Palm/ bamboo, Parquet/ polished wood, Vinyl/ asphalt strips, Ceramic tiles, Cement, Carpet/ rug, and others.	**Unimproved:** Earth/ sand, Dung, Wood planks, Palm/ bamboo, others Improved: Parquet/ polished wood, Vinyl/ asphalt strips, Ceramic tiles, Cement, Carpet/ rug	Unimproved Improved	= 0 = 1
Main wall material	No walls, Cane/ palm/ trunks, Dirt, Bamboo with mud, Stone with mud, Plywood, Cardboard, Reused wood, Metal/zinc, Cement, Stone with lime/ cement, Bricks, Cement blocks, Wood planks/ shingles, and others.	**Unimproved:** natural, no wall, cane/palm/trunks, dirt, rudimentary, bamboo with mud, Wood planks/ shingles, stone with mud, Plywood, Cardboard, Reused wood, Metal/zinc, and others. **Improved:** cement, Stone with lime/ cement, cement blocks, bricks	Unimproved Improved	= 0 = 1
Main roof material	No roof, Thatch/ palm leaf, Rustic mat, Palm/ bamboo, Wood planks, Cardboard, Metal/ zinc, Wood, Ceramic tiles, Cement, Roofing shingles, and others	**Unimproved:** natural, no roof, palm leaf, sod, rudimentary, rustic mat, bamboo, cardboard, wood planks **Improved:** cement, roofing shingles, ceramic tiles, Metal/ zinc, Ceramic tiles	Unimproved Improved	= 0 = 1

Each woman was assessed based on the re-categorized score in each of HHEC, thus, the maximum score attainable is 6. Consequently, this overall score was disaggregated into 4 categories as:

$$HEEC=\{\begin{array}{l} \mathrm{None}/\mathrm{unimproved}\;\mathrm{if}\;\mathrm{HHEC}\;\mathrm{total}\;\mathrm{score}\;\mathrm{is}\;0\\ \mathrm{Poor}/\mathrm{partially}\;\mathrm{improved}\;\mathrm{if}\;\mathrm{HHEC}\;\mathrm{total}\;\mathrm{score}\;\mathrm{is}\;1‐2\\ \mathrm{Fair}/\mathrm{averagely}\;\mathrm{improved}\;\mathrm{if}\;\mathrm{HHEC}\;\mathrm{total}\;\mathrm{score}\;\mathrm{is}\;3‐5\\ \mathrm{Complete}/\mathrm{improved}\;\mathrm{if}\;\mathrm{HHEC}\;\mathrm{total}\;\mathrm{score}\;\mathrm{is}\;6 \end{array}$$

Other independent variables include; Age, place of residence, region of residence, level of education, household wealth index, ethnicity, work status, parity, marital status and sex of household head.

The variable household wealth was created as a composite measure of a household's cumulative living standard using Principal Component Analysis (PCA). It was generated using data on a household’s ownership of selected assets as presented in the DHS guide [25–28]. However, some variables used in the creation of the original wealth index in DHS included the variables used in the calculation of household environmental conditions (source of drinking water, toilet facilities, flooring materials, wall materials, and roof materials). Therefore, such variables were excluded from the newly generated household wealth to avoid multi-collinearity effect on the emerging results from this study. Each household asset for which information is available was assigned a weight generated through PCA. Thereafter, the asset scores were standardized in relation to a standard normal distribution (*μ* = 0,*σ*^2^ = 1). Each household was assigned a standardized score for each asset, and the score differs subject to whether the household owned that particular asset or not. These scores are summed by households, and individuals are ranked according to the total score of the household in which they reside. The sample was divided into terciles of household wealth as poor, middle and rich.

### Data analysis

Due to the cluster design method used for sampling collection, the data was weighted before use. The weighting of the data becomes important in this situation in order to extrapolate and take into consideration other areas excluded in the clusters during the survey. Weights are adjustment factors applied to each case in tabulations to adjust for differences in probability of selection and interview between cases in a sample, due to cluster design used for the survey. During the survey, the sample was selected with unequal probability to expand the number of cases available (and hence reduce sample variability) for certain areas or subgroups for which statistics are needed. Also, the weighting of the data was done to adjust for the possible differences in response rates across the states of the federation. Sample weights were calculated to six decimals but are presented in the standard recode files without the decimal point. Therefore, for the data set analysed for this study, a weight variable was created by dividing the sampling weight by 1,000,000 before it was used to approximate the number of cases. In addition, because standard errors, confidence intervals and significance testing are required in our outputs, we took into consideration of the complex sample design by using cluster variable, stratification variable, and the weight variable to make adjustment as appropriate [27,29].

The data were analysed using descriptive statistics, Chi-square, and logistic regression model. Frequency distribution was used to present the data and Chi-square test was conducted to determine the association between HHEC and nutritional status. This type of association was also examined for other explanatory variables included in the study. Logistic regression was used due to the dichotomous nature of the dependent variable to identify the predictors of underweight among the subjects. At the level of multivariate analysis, five models were used to define the relationship between underweight and HHEC. SPSS version 20.0 and Microsoft Excel software was used for all analyses.

The variables included in each of the five models are as follows: Model 1 is the bivariate model that examines the relationship between HHEC and underweight while model 2 is a multivariate model that involves the dependent variable (BMI), HHEC and demographic variables (parity and age). Model 3 included only the dependent variable, HHEC and economic (household wealth, work status in the past 12 months before the survey) while the variables included in Model 4 are the dependent variable, HHEC, and social (region, residence, education, ethnicity, marital status and sex of the household head) explanatory variables. In the last model, all variables found to be statistically significant at the bivariate level were included in the model to identify the important predictors of underweight (α = .05).

The logistic regression model is of the form;

$${log}_{e}(ϴ/(1‐ϴ))=\xi_{0}+\xi_{1}x_{i1}+\xi_{2}x_{i2}+\cdots +\xi_{p}x_{ij};i=1,2,3,\ldots ,n$$

Where ϴ is the proportion of women who are underweight and *ξ_i_* are the regression parameters to be estimated with the exponential of *ξ* being the odds ratio and *x_ij_*, are the explanatory variables.

### Availability of data and materials

The data underlying the results presented in this study is a third-party data owned by the Demographic Health Survey (DHS) Program and is available to registered users from the MEASURE DHS website. Restrictions apply to the availability of the data which were used under license for the current study, and so are not publicly available. Data are however available upon reasonable request through https://dhsprogram.com/data/dataset_admin/login_main.cfm? Prospective users are expected to complete a user registration form in addition to providing a proposed project title and a 300 words (minimum) abstract describing how the user plan to use the DHS data. A link through which the requested data could be downloaded will be sent to the potential user within five days once approval has been granted. We the authors confirm that researchers can access the data set used in this study in the same manner as the authors and that the authors had no special access privileges to the data sets that other researchers would not have.

## Results

The distribution of the characteristics of the women sampled is presented in Table 2. About 40.0% of the women were aged 15–24 years while 23.8% were aged 25–34 years. Forty-two percent of the women had secondary education, while more than half were currently in union (57.2%) and lived in a rural area (52.2%) in Nigeria. Regarding work status, 60.9% are currently working. A greater proportion (78.0%) of the women had their homes headed by a male. About 18.6% and 53.5% of the respondents live in good and fair housing conditions respectively. Urbanization and recent preference for living in modern cutting edge housing over living in shelters that are basic in nature could probably explain why a larger proportion of the study participants resided in houses with fairly/average environmental conditions.

**Table 2:** Socio-demographic characteristics of mothers, NDHS, 2013.

Background characteristics	Number	Percent
*Age*		
15–24	9242	39.6
25–34	5565	23.8
35–49	8537	36.6
*Highest educational level*		
None	6965	29.8
Primary	4017	17.2
Secondary	9745	41.7
Higher	2617	11.2
*Marital status*		
Never in union	8496	36.4
Currently in union	13352	57.2
Formerly in union/living with a man	1496	6.4
*Place of residence*		
Urban	11169	47.8
Rural	12175	52.2
*Region*		
North Central	3425	14.7
North East	2993	12.8
North West	5767	24.7
South East	3240	13.9
South South	3498	15.0
South West	4421	18.9
*Ethnicity*		
Hausa/Fulani	6206	26.6
Igbo	4066	17.4
Yoruba	3912	16.8
Others	9160	39.2
*Household Wealth*		
Poor	6774	29.6
Middle	7697	33.7
Rich	8399	36.7
*Working status (Currently working)*		
No	9118	39.1
Yes	14227	60.9
*Parity*		
0	9802	42.0
1–2	3371	14.4
3–4	3751	16.1
5+	6420	27.5
*Sex of household head*		
Male	18219	78.0
Female	5125	22.0
*Household environmental conditions*		
None/unimproved	1735	7.4
Poor/partially improved	4783	20.5
Fair/averagely improved	12495	53.5
Complete/improved	4332	18.6

Table 3 presents the proportion of women who are underweight based on their socio-demographic characteristics. More women who are underweight are in the youngest age group 15–24 years (19.7%), had no formal education (14.9%), and are unmarried (18.1%) (p<0.001). Also, undernutrition was significantly higher among women who resided in the North Western (17.3%) part of Nigeria, lived in rural areas (13.3%), had never given birth to a child (18.2%), and whose households were headed by a male (12.2%). The prevalence of underweight differed between ethnic groups. The proportion of women who are underweight decreased with increasing wealth tercile (p<0.001).

**Table 3:** Maternal characteristics in relation to nutritional status.

Background characteristics	Underweight women (%)	Total number of women	X^2^ value	p-value
*Age*			948.60	<0.001
15–24	19.7	9242		
25–34	7.4	5565		
35–49	5.9	8538		
*Highest educational level*			216.86	<0.001
None	14.9	6965		
Primary	10.9	4017		
Secondary	11.8	9745		
Higher	4.2	2617		
*Marital status*			515.43	<0.001
Never in union	18.1	8496		
Currently in union	8.2	13352		
Formerly in union/living with a man	7.5	1496		
*Place of residence*			57.92	<0.001
Urban	10.1	11169		
Rural	13.3	12175		
*Region*			404.46	<0.001
North Central	8.0	3425		
North East	16.7	2993		
North West	17.3	5767		
South East	7.5	3240		
South South	10.2	3498		
South West	11.7	4421		
*Ethnicity*			479.16	<0.001
Hausa/Fulani	19.2	6206		
Igbo	7.1	4066		
Yoruba	10.0	3912		
Others	9.4	9160		
*Household wealth*			188.696	<0.001
Poor	15.5	6774		
Middle	12.4	7697		
Rich	8.4	8398		
*Working status (Currently working)*			615.70	<0.001
No	18.3	9118		
Yes	7.6	14227		
*Parity*			676.19	<0.001
0	18.2	9802		
1–2	7.6	3371		
3–4	6.4	3751		
5+	7.2	6420		
*Sex of household head*			16.85	<0.001
Male	12.2	18219		
Female	10.1	5125		

**Fig 2:**
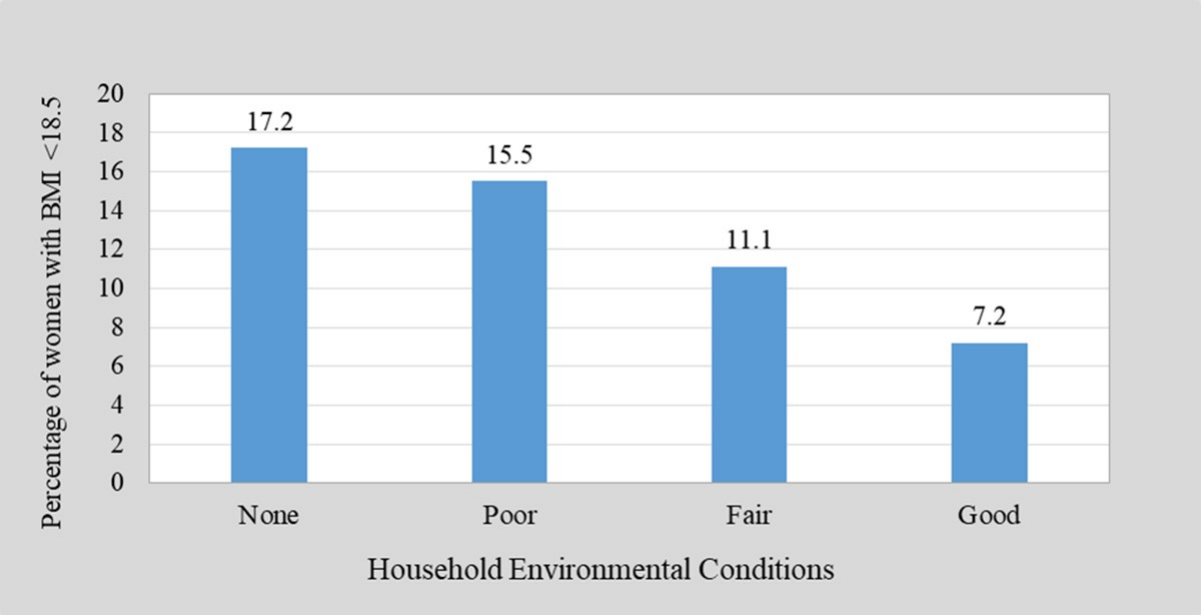
Distribution of underweight according to environmental conditions.

The distribution of underweight women according to HHEC (cooking fuel, toilet type, source of drinking water, and housing materials) is shown in Fig 2. The prevalence of underweight reduces with an increasing improvement in household environmental conditions. More women (17.2%) and (15.5%) who were underweight lived in houses with unimproved and partially improved HHEC respectively compared to those who lived in houses with improved (7.2%) HHEC.

### Multivariate results

For the multivariate analysis, five models were fitted while exploring the likely association between maternal undernutrition and exposure to household environmental conditions while other factors were controlled for as covariates (Table 4). The result indicated that household environmental conditions were significantly associated with underweight among surveyed women. In the first model, the odds of undernutrition was higher among women who lived in houses with unimproved HHEC (OR = 2.66; C.I = 2.25–3.15, p <0.001) and partially improved (OR = 2.34; C.I = 2.04–2.69) HHEC compared to those who lived in houses with improved environmental conditions. This association was consistent across models 2 to 4, even after adjusting for the effects of demographic, economic, and social factors. For example in model 2, the odds of maternal undernutrition was three times (aOR = 2.92, C.I = 2.45–3.48, p <0.001) as high among women living in a house with unimproved HHEC than those in houses with improved HHEC.

**Table 4:** Adjusted Odds Ratio for the relationship between nutritional status and Household environmental index, and other independent variables.

Background characteristics	Model 1	Model 2	Model 3	Model 4	Model 5
*Household environmental factors*					
None/unimproved	2.66 (2.25–3.15)*	2.92 (2.45–3.48)*	1.84(1.51–2.32)*	1.64 (1.33–2.03)*	2.02(1.37–2.97)*
Poor/partially improved	2.34 (2.04–2.69)*	2.52 (2.18–2.91)*	1.67(1.42–1.96)*	1.51 (1.26–1.80)*	1.72(1.20–2.46)*
Fair/averagely improved	1.59 (1.41–1.82)*	1.55 (1.36–1.77)*	1.37(1.19–1.57)*	1.19 (1.03–1.37)*	1.53(1.11–2.10)*
Complete/improved	1	1	1	1	1
*Age*					
15–24		3.54 (2.97–4.23)*			2.38(1.88–3.00)*
25–34		1.40 (1.20–1.64)*			1.35(1.13–1.61)*
35–49		1			1
*Parity*					
0		1.15 (0.96–1.39)			1.12(0.87–1.43)
1–2		0.62 (0.51–0.76)*			0.80(0.64–1.01)
3–4		0.81 (0.68–0.96)*			0.95(0.78–1.16)
5+		1			1
*Household wealth*					
Poor			1.58(1.39–1.79)*		2.14(1.69–2.71)*
Middle			1.41(1.25–1.57)*		1.68(1.35–2.10)*
Rich			1		1
*Working status (Currently working)*					
No			2.72(2.50–2.96)*		1.31(1.12–1.51)*
Yes			1		1
*Highest educational level*					
None				3.70 (2.90–4.71)*	3.13(1.79–5.45)*
Primary				3.66 (2.90–4.61)*	2.81(1.62–4.85)*
Secondary				2.45 (1.99–3.01)*	2.10(1.22–3.60)**
Higher				1	1
*Marital status*					
Never in union				5.03(4.52–5.60)*	2.38(2.05–2.74)*
Formerly in union/living with a man				1.27(1.01–1.57)*	1.36(1.09–1.69)*
Currently in union				1	1
*Place of residence*					
Urban				1	1
Rural				1.14 (1.03–1.27)*	1.07(0.88–1.30)
*Region*					
North Central				1	1
North East				2.11 (1.78–2.51)*	2.01(1.51–2.65)*
North West				1.72 (1.43–2.06)*	1.46(1.08–1.97)*
South East				1.24 (0.89–1.71)	1.94(0.99–3.81)
South South				1.15 (0.95–1.38)	1.29(0.90–1.85)
South West				1.60 (1.30–1.98)*	2.15(1.46–3.15)*
*Ethnicity*					
Hausa/Fulani				1	1
Igbo				0.42 (0.31–0.58)*	0.40(0.21–0.74)*
Yoruba				0.65 (0.52–0.83)*	0.47(0.31–0.70)*
Others				0.50 (0.44–0.58)*	0.57(0.46–0.69)*
*Sex of household head*					
Male				1.09 (0.97–1.22)	1.02(0.78–1.32)
Female				1	1

* Significant at 5% test of logistic regression coefficients significance.

In model 5 where all the factors were controlled for, the odds of undernutrition (aOR = 2.02, C.I = 1.37–2.97, p <0.001) were twice as high among women who lived in houses with no HHEC. This thus, suggests that HHEC is an important predictor of maternal undernutrition in Nigeria.

Overall, younger women (15–24 years) were more likely to be underweight (aOR = 2.38, C.I. = 1.88–3.00, p <0.001) than older women (35–49 years). Women who are of poor wealth status are more likely to be underweight (aOR = 2.14, C.I. = 1.69–2.71, p <0.001) than those in the highest wealth group while women who were never in a union were more likely to be underweight (aOR = 2.38, C.I = 2.05–2.74, p <0.001) than those currently in a union (Table 4). Other predictors of underweight among women include having a primary education, no occupation, and living in the North East region of Nigeria. All other ethnic groups had significantly lower odds of undernutrition compared to the Hausa group.

## Discussion

Adequate nutrition is important to the health of women and children. Undernutrition in women promotes susceptibility to infections, slow recovery from illness among other health outcomes. This study provided evidence on the importance of household environmental conditions in shaping the nutritional status of women of childbearing age in Nigeria.

Findings from this study established that living in a house with unimproved HHEC is a significant predictor of undernutrition in women as more women that were underweight lived in houses with unimproved HHEC. This association persisted even when the effects of demographic, economic, and social factors were adjusted for. In Nigeria, living in houses built with improved housing materials is protective against poor health outcomes while houses with poor housing characteristics can predispose to many illnesses [30]. Living in an unimproved housing predisposes one to more malaria episodes from mosquito bites than those living in improved housing conditions [31]. Anaemia is one of the nutritional complications of malaria infection and it is a determinant of maternal undernutrition [32]. Poverty is prevalent in Nigeria with many Nigerians living on less than a dollar per day [33] thus, hindering individuals and families from affording basic essential needs including living in houses with improved characteristics and environmental conditions. In Ethiopia, women who lived in unimproved houses had higher odds of maternal nutrition than those living in improved houses [34]. This gave credence to the findings of our study. In Jordan, the materials that constitute the wall, floor, and roof of a building were found to be a significant determinant of the health status of its occupants [35]. In Ethiopia, women who lived in a hut house (poorly constructed) were more likely to be malnourished than those living in a corrugated iron sheet house (properly constructed) [7].

Our findings corroborated the findings from previous studies that reported an association between maternal undernutrition and drinking water from unimproved sources [7], use of unclean fuel [22] and unimproved sanitation [36,37]. Access to improved drinking water, sanitation, and hygiene facilities is an indispensable element of a healthy community and play a positive impact on the nutritional status of the population [17,37]. The prevalence of undernutrition recorded among women in this study may be connected with the proportion of Nigerians who had access to improved water supply (58%) and improved sanitation facilities (37%) in 2013 [38]. Women who used water from unimproved sources were more likely to be underweight while access to safe water and adequate sanitation contribute to reduction in the prevalence of underweight [39]. Lack of access to improve drinking water and sanitation may predispose individuals to diarrhea [36,40], parasitic infections [23] helminth infections [41,42], which can affect the nutritional status of an individual. Helminths infections can cause a reduction in the desire for food, malabsorption of nutrients, growth impairment and anaemia [17,43]. Moreover, exposure to maternal smoking and biofuel smoke and the risk of anaemia has been documented in the literature [22].

As expected, this study further revealed that women in a household with poor socio-economic status have a higher probability of being undernourished. Socio-economic status is a predictor of underweight in developing countries. Poor wealth status contributes to undernutrition in women through poor food intake and increased exposure to infections [44]. With rising food prices, the recent recession of the economy, and the steady increase in inflation that has reduced the value of Nigerian currency, a substantial number of Nigerian households are becoming food insecure [45]. The effects of household food insecurity include protein-energy malnutrition, micronutrient deficiencies diet-related non-communicable diseases among others [45]. Studies have shown that mothers with poorest wealth status are more likely to give preference to their children by ensuring that their wards are well fed, while mothers embrace risky coping strategies including reducing food consumption patterns and compromising on the quality of their diet [46,47]. This can make mothers deficient in essential macro- and micronutrients [46,47].

Furthermore, women resident in North west Nigeria were more likely to be undernourished than women from the southern region of Nigeria. Similar findings have been documented in the year 2014 report on the nutrition and health situation of Nigeria [48]. The marked variance in the sociocultural factors across the geo-political zones in Nigeria, mostly between the North and South, could be a pointer to the high prevalence of undernutrition recorded among women from the North west [45]. Religious beliefs that allow a man to marry more than one woman in addition to high poverty in Northern Nigeria could cause reduced food intake per head thus contributing to undernutrition [44]. Also, the higher proportion of women in Northern Nigeria who use water from unimproved water sources is higher than women in the South. As described above, unimproved water has been associated with diarrhoea which causes undernutrition in women [44].

Other predictors of maternal undernutrition in this study include educational, marital and employment status. These findings are consistent with reports of studies from other locations which found associations between maternal undernutrition and not currently working, having primary education, and never been married [49,50].

Some limitations were identified in this study. Since the 2018 NDHS was conducted from 14 August 2018 to 29 December 2018, it is possible that the nutritional status of the study participants varied across the months of the year following food availability, intake, and disease pattern. Data collection that spans across the months of the year would give a better portrayal of nutritional status of reproductive age women.

## Conclusion

This study explored the role of household environmental conditions in the nutritional status of women of childbearing age in Nigeria. The lack of satisfactory household environmental conditions are among the key factors promoting malnutrition in women. Achieving the SDGs and other key global health goals, such as ending preventable child and maternal deaths, will equally involve tackling malnutrition in all its forms. The realisation of these goals will include educating the populace through advocacy on the importance of safe drinking water, practicing good sanitation and hygiene practices among other environmental conditions. There is a need for the government and other relevant stakeholders to formulate and implement the guidelines on improved housing to guide against the occurrence of a wide range of preventable nutrition-related poor health outcomes often associated with poor housing and environmental conditions.

The integration of household environmental and nutrition programmes could assist in addressing the burden of undernutrition among women of childbearing age in Nigeria. This will contribute significantly to achieving goals 1 to 3 of the SDGs, and the United Nations Decade of Action on Nutrition (2016–2025).
